# The first full study of heavy metal(loid)s in western-European hedgehogs (*Erinaceus europaeus*) from Portugal

**DOI:** 10.1007/s11356-024-31877-2

**Published:** 2024-01-16

**Authors:** Catarina Jota Baptista, Fernanda Seixas, José M. Gonzalo-Orden, Carla Patinha, Pedro Pato, Eduardo Ferreira da Silva, María Casero, Erica Brazio, Ricardo Brandão, Daniela Costa, Teresa L. Mateus, Ana C. Coelho, Paula. A. Oliveira

**Affiliations:** 1 Department of Veterinary Sciences, School of Agrarian and Veterinarian Sciences (ECAV), University of Trás-os-Montes and Alto Douro (UTAD), Vila Real, Portugal; 2 Centre for the Research and Technology of Agro-Environmental and Biological Sciences (CITAB-Inov4Agro), UTAD, Vila Real, Portugal; 3 Institute of Biomedicine (IBIOMED), University of León, León, Spain; 4 Faculty of Veterinary Medicine, Egas Moniz Center for Interdisciplinary Research (CiiEM); Egas Moniz School of Health &Science, Almada, Portugal; 5 Animal and Veterinary Research Centre (CECAV), UTAD, Associate Laboratory for Animal and Veterinary Sciences (AL4AnimalS), Vila Real, Portugal; 6 GEOBIOTEC & Department of Geosciencie, University of Aveiro, Aveiro, Portugal; 7 RIAS-ALDEIA - Wildlife Rehabilitation and Research Centre, Ria Formosa Natural Park, Olhão, Portugal; 8 Lisbon Wildife Rescue center (LxCRAS), Forest park of Monsanto, Lisbon, Portugal; 9 CERVAS-ALDEIA – Centre of Ecology, Wild animals’ Rehabilitation and Surveillance, Gouveia, Portugal; 10 CISAS-Centre for Research and Development in Agrifood Systems and Sustainability, Superior Agrarian School, Polytechical Institute of Viana do Castelo, Viana do Castelo, Portugal; 11 EpiUnit – Public Health Institute of University of Porto, Laboratory for Integrative and Translational Research in Population Health, Porto, Portugal

**Keywords:** environmental contamination, One Health, trace elements, wildlife

## Abstract

The western-European hedgehog (*Erinaceus europaeus*) is an insectivore with a wide distribution in Portugal and a potential tool for biomonitoring relevant One Health hazards, including heavy metal(loid)s’ pollution. The aim of this study was to positively contribute to the current knowledge about the metal(loid) pollution in Portugal. Forty-six hedgehogs (from rescue centres; with known provenance) were necropsied. Sex, age category and weight were determined. Spines, liver and kidney were collected, and metalloid concentrations were determined by inductively coupled plasma mass spectrophotometry (ICP-MS). In general, results did not present alarming metal(loid) concentrations, with the exception of cadmium (Cd) (in the kidneys) and copper (Cu). Hedgehogs from Viana do Castelo and Viseu showed elevated concentrations of arsenic (As) and Castelo Branco presented concerning values of cadmium (Cd). Adult and heavier hedgehogs tended to present higher levels of metal(loid)s. Sex does not seem to significantly affect the metal(loid)s’ concentrations. Further analysis would be needed to prioritize areas with detail and allow the application of the necessary mitigation strategies.

## Introduction

Heavy metal(loid)s are a group of toxic chemical substances that interfere with different physiological body functions after acute or chronic exposure. Metal(loid)s can be considered essential (as Cr or Cu, which participate in some biological processes) and non-essential (as Cd or Pb, which have no biological function) (Ali and Khan 2019). Nevertheless, both can be toxic for living organisms, depending on the doses or frequency of exposure. For instance, Pb is considerably hepatoxic, Cd is highly nephrotoxic, and As is carcinogenic (especially in its inorganic form) (Ali et al. 2019; Balali-Mood et al. 2021). Moreover, metal(loid)s have the potential to be perpetuated in the environment (without being degraded), bioaccumulate in trophic chains (including second and third-level consumers) and accumulate in biological tissues (Ali and Khan 2019; Khan et al. 2015).

Western-European hedgehogs (*Erinaceus europaeus*) are insectivorous mammals with a broad distribution in most European countries. Although its population had been considered stable in the Iberian Peninsula in the past, recent national reports have been presenting a population decline in several European countries, suggesting vulnerability of these populations (Amori 2016, App et al. 2022; Haigh et al. 2012; Pettett et al. 2018; Mathews and Harrower 2020; Taucher et al. 2020; Williams et al. 2018). Despite these aspects (food regimen and distribution), they are resilient and well adaptable to different habitat types (Berger et al. 2020; Gazzard et al. 2022; Rasmussen et al. 2019a). Therefore, *E. europaeus* has been used to study and biomonitor different One Health hazards such as zoonotic diseases (Hofmannová and Juránková 2019; Jota Baptista et al. 2021; Thamm et al. 2009), environmental pollution (D’Havé et al. 2006; Dowding et al. 2010; Rautio et al. 2010; Vermeulen et al. 2009) or antibiotic resistance (Di Francesco et al. 2020; Jota Baptista et al. 2021; Larsen et al. 2022; Rasmussen et al. 2019b), in different geographical regions. Hedgehogs (as well as other insectivores and small mammals) have been used in different European countries to assess heavy metal(loid) pollution, providing important information regarding public health and nature conservation (D'Havé et al. 2005; D’Havé et al. 2006; Pankakoski et al. 1993a; Rautio et al. 2010; Sánchez-Chardi et al. 2007; Sánchez-Chardi and López-Fuster 2009; Vermeulen et al. 2009). Furthermore, the liver and kidneys have been largely used as invasive samples in several biomonitoring studies. They frequently accumulate large amounts of these substances over the years and participate in metabolism, detoxification, and elimination (Baptista et al. 2022; Jota Baptista et al. 2022; Sánchez-Chardi et al. 2009). On the other hand, hair and spines have been recently used as a non-invasive alternative sample in several species, since significant correlations have often been found with internal metal(loid) concentrations (D’Havé et al. 2006; Hernout et al. 2016; McHuron et al. 2019; Nielsen et al. 1994; Vermeulen et al. 2009).

Notwithstanding, there is a lack of biomonitoring studies in Portugal using wildlife mammals. Therefore, this study aims (1) to assess metal(loid)s concentrations using the liver, kidney and spines of *E. europaeus*; (2) to identify correlations between those organs (namely between spines and the internal tissues, which can be used to design new non-invasive studies); (3) to find associations between metal(loid) concentrations and clinical or biological data (as age, sex or geographical provenance) and, at the end, (4) to help characterize the heavy metal(loid) pollution problem for micromammalian biodiversity in Portugal.

## Materials and methods

### Necropsies and sampling

All the hedgehogs considered in this study died in one of three different Portuguese rescue centres (CERVAS, from the north; LxCRAS from the centre and RIAS, from the south of Portugal) or were found dead in the wild. The reason for admission (at the rescue centre) and provenance was also registered. None of them was killed for study purposes. These animals were euthanized according to the rescue centre’s internal policy or died naturally, between 2019 and 2021. All the included hedgehogs spent less than 5 days at the rescue centre. Therefore, no ethical approval was needed to perform this study. A full necropsy was performed on 46 hedgehogs. Carcasses were weighted (g); sex and age categories (hoglets or pre-weaned, juveniles or post-weaned and adults) were estimated according to the published literature (Bexton and Robinson 2003). Dorsal skin (with spines), liver and kidney (2–10 g) were collected in zip bags and stored under − 10 °C until analysis.

### Drying process and metal(loid) determination

The day before lyophilisation, kidney and liver samples were transferred to a − 20 °C freezer. Then, they were completely freeze-dried for 48 h at − 56 °C (LaboGene CoolSafe®). The weight of each sample was recorded before and after lyophilisation (Kern ALT® precision scale), in order to determine the humidity of the tissue, removed during the lyophilisation (average values of 75.1% for the liver and 74.2 % for the kidney).

Spines were carefully removed from the attached skin with disinfected tweezers and weighted (1–2 g) in a graduated glass. Then, these glasses were filled with deionised water and washed in an ultrasound machine (Sonorex RK 106®) for a complete cycle (15 min). Spines were removed from each glass (using disinfected tweezers, to avoid cross-contamination) and placed in Petri plates. Then, all these Petri plates were dried overnight (55 °C) in the oven.

**Fig. 1:**
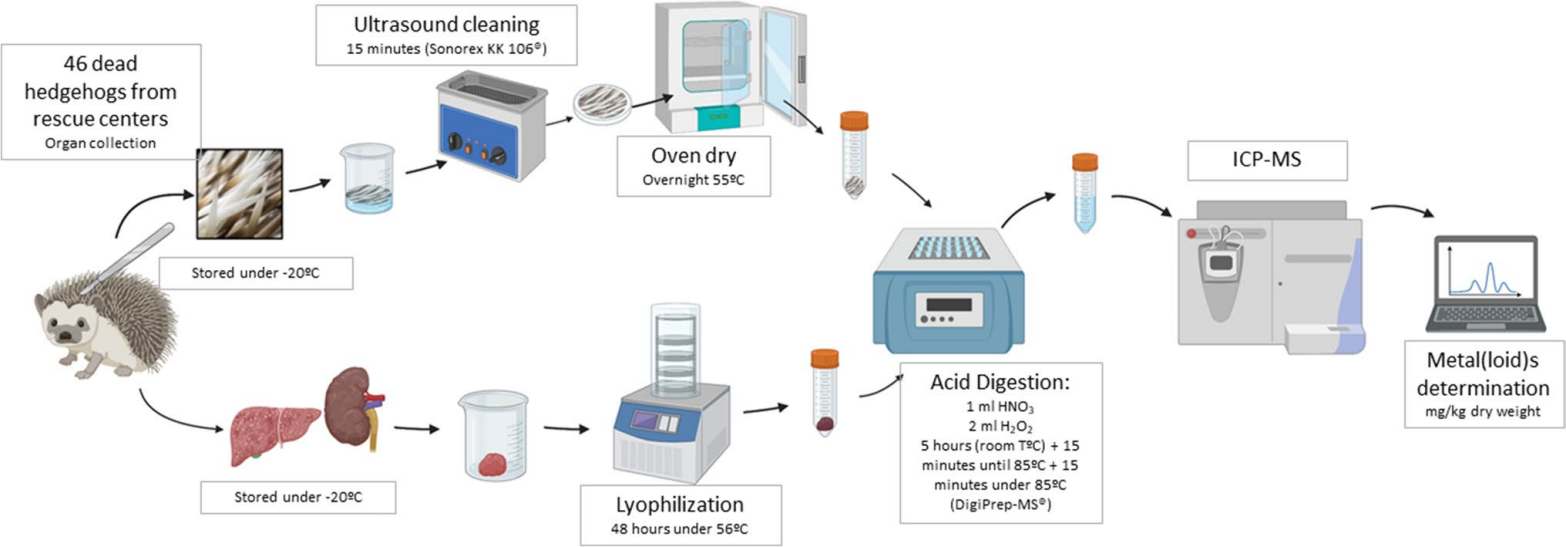
Diagram of the methodology (created with BioRender.com)

Approximately 0.5 g of each dried sample (spines, liver and kidney) was weighted on the precision scale and transferred to digestion tubes. Then, 1 ml of HNO_3_ was added to each tube and left at room temperature overnight. Then, 2 ml of H_2_O_2_ was added to each sample. After 5 h under room temperature, samples were placed on a digestion plate (DigiPrep-MS®), where the temperature increased progressively for 15 min until reaching 85 °C. Then, they remained at this temperature for another 15 min. All the samples presented no visible solid particles after this digestion procedure. Concentrations of As, Cd, Cr, Co, Cu, Ni and Pb were determined by Agilent 7700 inductively coupled plasma mass-spectrophotometry (ICP-MS) (Agilent Technologies®, Santa Clara, CA, USA) in Geochemistry laboratory of Geosciences Department, Aveiro University. The selection of these elements was based in several criteria including the suitability of the available laboratory method to their determination, their importance under an animal health perspective, their relevance in terrestrial ecosystems and their presence in the Earth crust in this territory (Inácio et al. 2008). The whole methodology is illustrated in Fig. 1. A quality control of the mentioned procedures was applied to this methodology, including the use of certified reference materials (ERM BB185® and ERMDB001®, respectively for internal organs and spines), blank tubes and duplicates. The results were accepted when recoveries ranged between 70 and 120%. Average quantification limits (AQL) for each metal(loid) were 0.0125 mg kg^−1^ for As, 0.005 mg kg^−1^ for Cd, 0.0025 mg kg^−1^ for Co, 0.005 mg kg^−1^ for Cr, 0.005 mg kg^−1^ for Cu, 0.005 mg kg^−1^ for Ni and 0.005 mg kg^−1^ for Pb. Values below the quantification limits were presumed as zero.

### Statistical analysis

For all descriptive analysis and statistical tests, the IBM SPSS® Statistics 27 was used. Normality tests were applied to all the quantitative data (Shapiro-Wilk and Kolmogorov-Smirnov tests), revealing non-normal distributions. Correlations between metal(loid)s and body weight were calculated with the Spearman correlation test. Nonparametric linear regression was applied between spines and both soft tissues (liver and kidney). Mann-Whitney (two groups) and independent Kruskal-Wallis (more than two groups) tests were applied to all the samples according to the age group, sex, location and reason for admission. Bonferroni’s test was performed as a post hoc test, after the Kruskal-Wallis analysis, if applicable. A multiple non-parametric linear regression model (with variables’ ranks) was used to evaluate if spines’ determinations can be used to predict liver and kidney values. A critical *p* value of 0.05 was considered for all the statistical tests.

## Results

### Metal determinations in the tissues

Table 1 provides a summary (mean, standard deviation [SD], minimum and maximum values) of the seven metal(loid) concentrations in the three different analysed tissues. With exception of As, most metal(loid)s show statistically significant differences between the three analysed tissues (liver, kidney and spines).

**Table 1:** Summary of metal(loid)s’ concentrations in each tissue (liver, kidney and spines) (mg kg^−1^ dry weight)

	Liver (*N* = 42)			Kidney (*N* = 43)			Spines (*N* = 46)		
Metal(loid)	Mean ± SD	Min	Max	Mean ± SD	Min	Max	Mean ± SD	Min	Max
As	0.135 ± 0.142 *	0.000	0.694	0.143 ± 0.151 *	0.000	0.653	0.222 ± 0.240 *	0.032	1.017
Cd	0.953 ± 1.453 *	0.000	6.070	3.495 ± 6.421 *	0.000	33.555	0.013 ± 0.013 #	0.000	0.048
Co	0.267 ± 0.275 *	0.053	1.659	1.042 ± 0.973 #	0.120	3.515	0.046 ± 0.055 &	0.000	0.262
Cr	0.120 ± 0.113 *	0.019	0.695	0.225 ± 0.344 *	0.027	1.824	0.269 ± 0.292 #	0.052	1.865
Cu	35.66 ± 19.65 *	12.11	102.91	24.74 ± 21.05 #	6.50	149.01	9.94 ± 3.72 &	5.22	28.45
Ni	0.043 ± 0.073 *	0.006	0.479	0.241 ± 0.954 #	0.000	6.332	0.146 ± 0.207 #	0.000	0.937
Pb	0.673 ± 1.117 *	0.094	6.234	0.455 ± 0.951 #	0.021	6.235	0.481 ± 0.747 *#	0.037	4.202

Different symbols refer to statistically different values between the three tissues, according to the Independent Kruskal-Wallis test and Bonferroni post hoc test

### Correlations between metal(loid) concentrations and the body weight

These hedgehogs presented a mean body weight of 345.93 ± 233.19 grams (g), considering all age groups. The Spearman correlation test revealed several significant correlations between body weight and some metal(loid) determinations. In the liver, significant coefficients were found for As (0.495; *p* = 0.001), Cd (0.705; *p* < 0.001) and Co (0.542; *p* < 0.001). Considering the kidney, most metal(loid)s presented significant correlations: As (0.364; *p* = 0.018), Cd (0.763; *p* < 0.001), Co (0.492; *p* = 0.001), Cr (− 0.426; *p* = 0.005), Cu (− 0.346; *p* = 0.025). Finally, for the spines, this was only verified for As (0.348; *p* = 0.019) and Co (0.376; *p* = 0.011).

### Associations with age and sex

Significant differences between males and females were found only for the values of Pb in the liver (*p* = 0.03). Considering age, Table 2 summarizes differences between hoglets (or pre-weaned [H]), juveniles (or post-weaned [J]) and adults (A) that were found for some trace elements in different organs.

**Table 2:** Metal(loid) determinations across age groups (H, J and A); results from independent Kruskal-Wallis (IKW) test and Bonferroni’s post hoc (Bph) test (adjusted)

Organ	Metal(loid)	Mean ± SD (*n*)	*p* value IKW*	*p* value Bph**
Liver	As	H 0.076 ± 0.097 (*n* = 11) J 0.074 ± 0.046 (*n* = 9) A 0.190 ± 0.166 (*n* = 22)	**0.004**	(H-J) 1.000 (J-A) 0.104 **(H-A) 0.021**
	Cd	H 0.148 ± 0.279 (*n* = 11) J 0.103 ± 0.107 (*n* = 9) A 1.703 ± 1.686 (*n* = 22)	**0.000**	(H-J) 1.000 **(J-A) 0.004** **(H-A) < 0.001**
	Co	H 0.163 ± 0.113 (*n* = 11) J 0.147 ± 0.087 (*n* = 9) A 0.367 ± 0.342 (*n* = 22)	**0.004**	(H-J) 1.000 **(J-A) 0.042** **(H-A) 0.040**
	Cr	H 0.155 ± 0.187 (*n* = 11) J 0.073 ± 0.042 (*n* = 9) A 0.121 ± 0.067 (*n* = 22)	0.099	-
	Cu	H 30.95 ± 10.20 (*n* = 11) J 29.75 ± 12.58 (*n* = 9) A 40.44 ± 24.36 (*n* = 22)	0.564	-
	Ni	H 0.035 ± 0.017 (*n* = 11) J 0.077 ± 0.151 (*n* = 9) A 0.028 ± 0.018 (*n* = 22)	0.551	-
	Pb	H 0.467 ± 0.340 (*n* = 11) J 0.409 ± 0.349 (*n* = 9) A 0.884 ± 1.496 (*n* = 22)	0.554	-
Kidney	As	H 0.069 ± 0.103 (*n* = 11) J 0.108 ± 0.095 (*n* = 9) A 0.192 ± 0.173 (*n* = 23)	**0.036**	(H-J) 1.000 (J-A) 0.063 (H-A) 1.000
	Cd	H 0.060 ± 0.067 (*n* = 11) J 0.248 ± 0.380 (*n* = 9) A 6.408 ± 7.719 (*n* = 23)	**0.000**	(H-J) 1.000 **(J-A) 0.001** **(H-A) < 0.001**
	Co	H 0.504 ± 0.212 (*n* = 11) J 0.535 ± 0.584 (*n* = 9) A 1.497 ± 1.094 (*n* = 23)	**0.004**	(H-J) 1.000 **(J-A) 0.024** (H-A) 0.083
	Cr	H 0.395 ± 0.549 (*n* = 11) J 0.262 ± 0.394 (*n* = 9) A 0.129 ± 0.088 (*n* = 23)	0.244	-
	Cu	H 37.32 ± 38.93 (*n* = 11) J 20.68 ± 5.59 (*n* = 9) A 20.30 ± 6.28 (*n* = 23)	0.270	-
	Ni	H 0.099 ± 0.078 (*n* = 11) J 0.766 ± 2.088 (*n* = 9) A 0.107 ± 0.088 (*n* = 23)	0.571	-
	Pb	H 0.328 ± 0.392 (*n* = 11) J 0.270 ± 0.210 (*n* = 9) A 0.588 ± 1.265 (*n* = 23)	0.728	-
Spines	As	H 0.091 ± 0.064 (*n* = 12) J 0.262 ± 0.284 (*n* = 10) A 0.270 ± 0.259 (*n* = 24)	**0.005**	(H-J) 0.053 (J-A) 1.000 **(H-A) 0.004**
	Cd	H 0.011 ± 0.010 (*n* = 12) J 0.016 ± 0.018 (*n* = 10) A 0.013 ± 0.013 (*n* = 24)	0.912	-
	Co	H 0.023 ± 0.027 (*n* = 12) J 0.036 ± 0.043 (*n* = 10) A 0.061 ± 0.065 (*n* = 24)	0.075	-
	Cr	H 0.183 ± 0.155 (*n* = 12) J 0.247 ± 0.148 (*n* = 10) A 0.321 ± 0.374 (*n* = 24)	0.215	-
	Cu	H 7.75 ± 1.63 (*n* = 12) J 9.75 ± 1.84 (*n* = 10) A 11.12 ± 4.53 (*n* = 24)	**0.009**	(H-J) 0.096 (J-A) 1.000 **(H-A) 0.021**
	Ni	H 0.070 ± 0.059 (*n* = 12) J 0.178 ± 0.251 (*n* = 10) A 0.170 ± 0.231 (*n* = 24)	0.348	-
	Pb	H 0.238 ± 0.151 (*n* = 12) J 0.425 ± 0.351 (*n* = 10) A 0.626 ± 0.990 (*n* = 24)	0.276	-

Statistically significant differences are highlighted in bold

*H* hoglets (or pre-weaned), *J* juveniles (or pos-weaned), *A* adults

*Independent Kruskal Wallis test *p* value; **Bonferroni’s post hoc adjusted *p* value

Bold emphasis represents the statistically significant *p* values

### Associations with geographical location

**Fig. 2:**
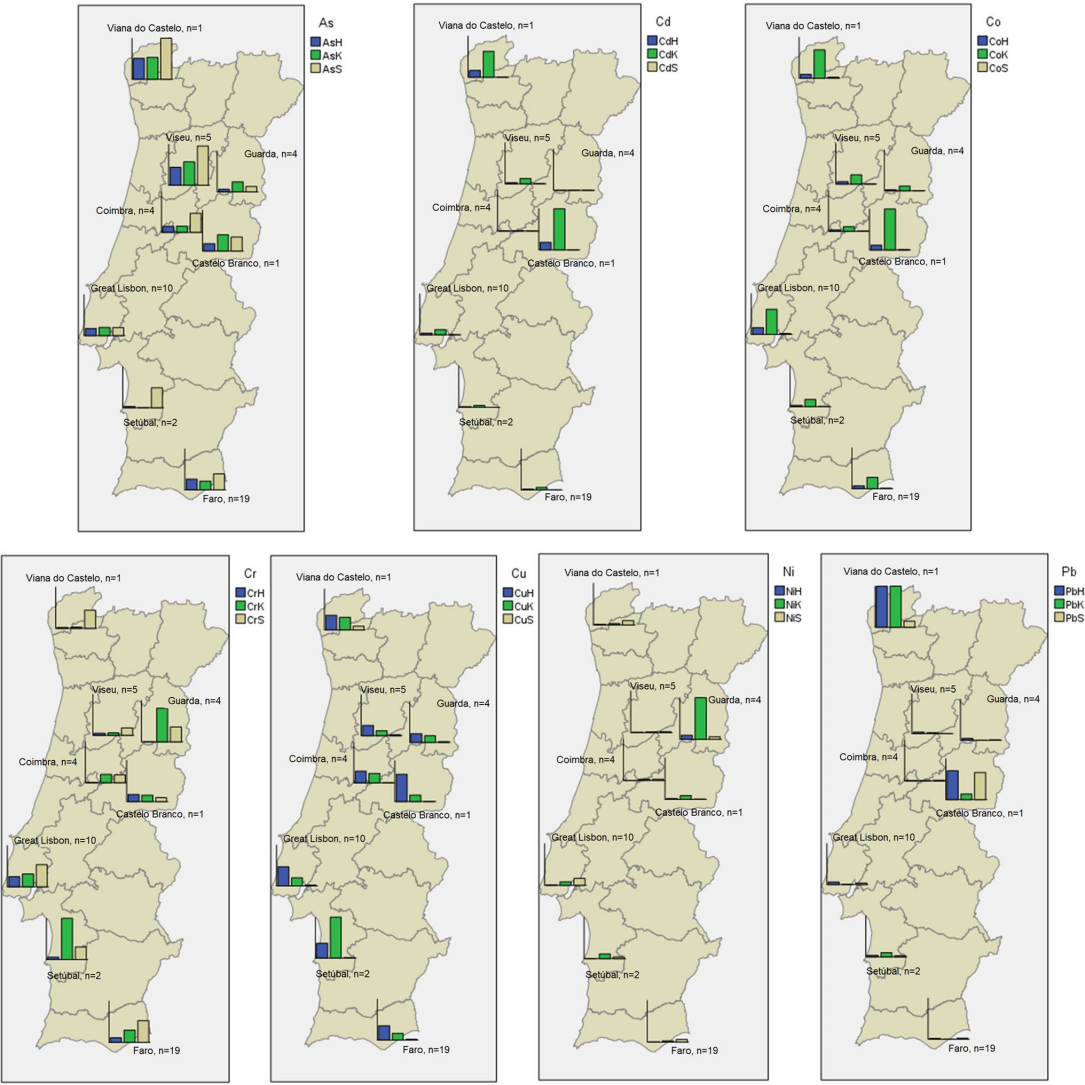
Distribution per district and per tissue (H liver; K kidney; S spines) of the mean values of seven metal(loid)s analysed in *E. europaeus*. The sampled regions are those that have a bar chart

Most metal(loid)s show different distributions across the Portuguese territory, from eight regions (from north to south; west to east): Viana do Castelo (*n* = 1); Viseu (*n* = 5); Guarda (*n* = 4); Coimbra (*n* = 4); Castelo Branco (*n* = 1); Great Lisbon (*n* = 10); Setúbal (*n* = 2) and Faro (*n* = 19). The following maps (Fig. 2) allow a complete illustration and summary of the differences between regions across the country, and also between tissues.

### Regression between spines and internal tissues

A multiple linear non-parametric regression showed that spines’ metal(loid) determinations cannot always predict liver and kidney values. Only Co and Ni presented statistically significant values of the regression coefficients between spines and one or both internal tissues. Table 3 summarizes the results obtained for the different metal(loid)s.

**Table 3:** Results of linear non-parametric regression between spines and internal tissues (kidneys and liver)

Metal(loid) in spines (independent variable)	Target (dependent variable)	*R*	*R*^2^	Coefficients		ANOVA	
				*t*	*p* value	*F*	*p* value
As (AsS)	Liver (AsH)	0.250	0.063	1.636	0.110	2.677	0.110
	Kidney (AsK)	0.245	0.060	1.618	0.113	2.618	0.113
Cd (CdS)	Liver (CdH)	0.253	0.064	1.651	0.107	2.725	0.107
	Kidney (CdK)	0.290	0.084	1.942	0.059	3.770	0.059
Co (CoS)	Liver (CoH)	0.771	0.594	**7.656**	**< 0.001**	**58.697**	**< 0.001**
	Kidney(CoK)	0.560	0.313	**4.323**	**< 0.001**	**18.692**	**< 0.001**
Cr (CrS)	Liver (CrH)	0.155	0.024	0.989	0.329	0.978	0.329
	Kidney(CrK)	0.087	0.008	0.560	0.579	0.314	0.579
Cu (CuS)	Liver (CuH)	0.302	0.091	2.002	0.052	4.010	0.052
	Kidney(CuK)	0.116	0.013	0.745	0.461	0.555	0.461
Ni (NiS)	Liver (NiH)	0.062	0.004	− 0.393	0.697	0.154	0.697
	Kidney(NiK)	0.340	0.115	**2.284**	**0.028**	**5.215**	**0.028**
Pb (PbS)	Liver (PbH)	0.134	0.018	0.855	0.397	0.732	0.397
	Kidney (PbK)	0.206	0.042	1.349	0.185	1.820	0.185

The underlined values indicate significant differences between spines and internal tissues

## Discussion

Livers, kidneys and spines from *E. europaeus* were used to characterize this animal exposure to heavy metal(loid)s in Portugal, with the aim of helping to describe this pollution type in this country and its impact on Portuguese fauna.

### Metal(loid)s concentrations in the tissues

A different distribution of the metal(loid)s was evident between different tissues, although not all of them. Similar findings have been reported by D'Havé et al. (2005); D’Havé et al. (2006). Particularly for As, no differences were found between the three tissues, which also happened to Rautio et al. (2010).

Globally, the present study revealed low values of As in the liver (AsH) (0.135 ± 0.142 mg kg^−1^ dry weight [dw]) and kidneys (AsK) (0.143 ± 0.151 mg kg^−1^ dw), when compared with other studies in hedgehogs (AsH, 0.45 ± 0.02 mg kg^−1^ dw; AsK, 0.47 ± 0.02 (Rautio et al. 2010) or AsH, 0.69 ± 0.13 mg kg^−1^ dw; AsK, 0.58 ± 0.07 (D'Havé et al. 2005; D’Havé et al. 2006)). Published reference values from small mammals (mice, rats, and voles) suggested that Cd values in the liver should range from 0.2 to 1.5 mg kg^−1^ dw, while Cd in the kidneys should range from < 0.1 to 5.6 mg kg^−1^ (Cooke 2011). Thus, the mean values of Cd obtained in this study (0.953±1.453 mg kg^−1^ in the liver; 3.495 ± 6.421 mg kg^−1^ in the kidneys) fit in these value ranges. Nevertheless, the maximum values and the SD demonstrate that some of our hedgehog samples showed high values of Cd.

Considering the analysed essential elements (as Cu or Co), the literature is frequently scarce regarding the effects of excessive amounts. Nevertheless, they are needed for some physiological reactions, generally in small quantities. For instance, Co is part of cobalamin, an enzyme essential to humans and most mammal species (Ertl et al. 2016). Regarding Cu, it is a microelement with an essential enzymatic role in human and animal’ metabolism (e.g. forming metalloenzymes), and it is usually found in higher concentrations compared to other microelements (Mccall et al. 2000; Ertl et al. 2016). For Co, the only values of Co found in literature for hedgehogs were 0.40 ± 0.04 mg kg^−1^ dw for the liver, 0.13 ± 0.03 mg kg^−1^/dw for the spines and 0.99 ± 0.32 mg kg^−1^ dw for the kidney (D'Havé et al. 2005; D’Havé et al. 2006). In the kidney samples studied, Co values were higher (1.042 ± 0.973 mg kg^−1^ dw). Considering Cr, the liver levels presented in this study were considerably lower (0.120 ± 0.113 mg kg^−1^), in comparison to other studies in hedgehogs (3.9 ± 0.2 mg kg^−1^ dw; D'Havé et al. 2005; D’Havé et al. 2006) or shrews (3.00 ± 0.48 mg kg^−1^ dw; Sánchez-Chardi et al. 2009). The same happened with the kidney (CrS) (0.225 ± 0.344 mg kg^−1^ dw) and spines’ levels (CrS) (0.269 ± 0.292 mg kg^−1^), considerably lower to that reported by D’Havé et al. (2006) (CrK, 3.4 ± 0.2 mg kg^−1^ dw; CrS, 4.3 ±0.3 mg kg^−1^ dw).

High levels of Cu were found in the kidney (24.74 ± 21.05 mg/kg dw) and liver (35.66 ± 19.65 mg/kg dw), with some animals passing 100 mg kg^−1^ dw, which is a high value for insectivores (D’Havé et al. 2006). Regarding Ni, the kidneys presented the higher values (0.241 ± 0.954 mg/kg mg kg^−1^ dw, followed by spines (0.146 ± 0.207 mg kg^−1^ dw) and then the liver (0.043 ± 0.073 mg kg^−1^ dw), which is the same order reported by Rautio et al. (2010). For Pb, the present study showed a mean value of 0.54 ± 0.70 mg kg^−1^ dw. Lead values in mammals are considered very variable, depending on the species and even between different populations of the same species. Mean values of 3.3 mg kg^−1^dw were obtained (and not considered as toxic) in mice and voles (Ma 2011).

The authors of the present work have hypothesized that these amounts of metal(loid)s (with the exception of As) may be causing biliary hyperplasia (i.e. an abnormal growth of the cells of the biliary ducts), already reported in other mammals as a cause of exposure to high quantities of these elements (Jota Baptista et al. 2023).

### Effects of body weight, age and sex

Correlation analysis demonstrated significant correlations between body weight and the levels of certain metal(loid)s in the studied tissues, especially the liver, in which all three significant correlations (As, Cd and Co) presented a *p* ≤ 0.001, and the kidneys, in which all the metal(loid)s (except Ni and Pb) showed significant correlations (*p* ≤ 0.025). In contrast, for the spines, this was only verified for As (0.348; *p* = 0.019) and Co (0.376; *p* = 0.011).

Hedgehogs are hibernators, showing more behaviour feeding right before hibernation and considerable flotations of body weight during the year. In theory, heavier and older hedgehogs were exposed to these compounds during more time (their lifetime) and consumed higher amounts of invertebrates, such as insects and earthworms. According to the literature, insectivores accumulate more metal(loid)s due to their food regimen, in comparison to other small mammals with similar body weight. In fact, it has been pointed as the main route of entrance of some metal(loid)s in the food chain (Rautio et al. 2010; Reinecke et al. 2000; Schrögel and Wätjen 2019). This aspect may explain the strong and mostly positive correlations found between body weight and metal(loid) levels, predominantly in internal tissues. Hibernation can be seen as a mechanism of protection against toxic metal(loid)s. Some are bound to proteins (as Cd) and, during the winter, the body burden is not increased. Active mammals during winter increase their food intake, intensifying their exposure to these compounds (Rautio et al. 2010). On the other hand, during hibernation, lipophilic compounds (as pollutants) may be slowly metabolized as the fat tissue is consumed for energy, increasing the serum levels (Florant 1998).

Moreover, adult hedgehogs presented higher levels for practically all the metal(loid)s, with significant differences for AsH, CdH, CoH, AsK, CdK, AsS and CuS. Heavy metal(loid)s are stable substances that remain unaltered in soils, water, plants and other organisms (Ali et al. 2019). Longer contact of older animals during their life with a contaminated environment (food, air and surfaces) may justify these results. Other studies have also documented this phenomenon (Rasmussen et al. 2023; Rautio et al. 2010). Age-related accumulation has been mentioned in the literature for other insectivores, such as moles (*Talpa europaea*) (Komarnicki 2000; Pankakoski et al. 1993b), and other small mammals, as grey squirrels (*Sciurus carolinensis*) (Hillis and Parker 1993; McKinnon et al. 1976; Wren 1986) and beavers (*Castor canadensis*) (Hillis and Parker 1993). In some cases, the difference can be colossal. For instance, Cd can be 3 and 15 times higher in the liver and kidneys, respectively, in adult moles, compared with juveniles (Pankakoski et al. 1993b).

In contrast, statistically significant differences between females and males were found only for Pb in the liver. Similarly, Rautio et al. (2010) suggested no effect of sex in heavy metal(loid) concentrations in hedgehogs.

### Associations with geographical location

Regarding the geographical distribution, the results suggested a very discrepant distribution of these environmental pollutants across the Portuguese territory, mainly justified by local and specific characteristics of each region. As previously mentioned, hedgehogs’ habitats are very diverse, including urban, suburban, rural and natural areas. Thus, they may be in contact with several sources of metal(loid) contamination of the soils and water (*e.g*. industrial, agricultural, mining, among others). Despite the economic vital importance of all these anthropogenic activities, the consequent residual and waste material represents the central cause of the high levels of certain elements detected in each location.

A study that analysed road dust samples from Viana do Castelo presented extremely high levels of As in suburban areas (180 mg kg^−1^), related to the fossil fuel combustion and agricultural activities developed in this region, representing a significant health risk (Candeias et al. 2020). This may explain the high values of As found in the present study in hedgehogs from Viana do Castelo, compared to other regions. Nevertheless, these authors admitted that the composition of dust presents enormous seasonal and geographical variations, which impairs the creation of a chemical fingerprint (Candeias et al. 2020). Possibly, future biomonitoring studies, using species (as hedgehogs) that accumulate these particles during all their lifetime, may provide a better perception of the long-term pollution by these compounds in Viana do Castelo.

A study comparing Viseu and Lisbon highlighted that the Portuguese capital is enriched in elements of anthropogenic origin, while in Viseu, these compounds have mainly a geogenic origin (Cachada et al. 2013). In that study, Co was found in elevated concentrations and presented a high potential available fraction in Lisbon, especially in the city centre (Cachada et al. 2013), a pattern that is also easily seen in the hedgehogs’ results.

Hedgehogs from Setubal district seem to be exposed to high levels of Cr and Cu. A study in Seixal (located in Setubal district) suggested that these high levels can be attributed to industrial activity (namely steelworks) and the road traffic, respectively (Abecasis et al. 2022). Similarly, in Seixal bay, Cr was one of the most common elements detected in suspended particulate matter of the estuary, while Cu was frequently and mostly detected in the bottom sediments (Caçador et al. 2012). Moreover, hedgehogs from southern Portugal (including Faro district) showed generally lower concentrations of As and Pb in comparison with the northern parts of the country, which is in agreement with soil geochemical atlas of the Portuguese territory published by Inácio et al. (2008).

Pereira et al. (2006) performed a biomonitoring study using the Algerian mice (*Mus spretus*) and wild rats (*Rattus rattus*) in a geographical region not assessed in the current study: the abandoned mine area of São Domingos in East Alentejo (South Portugal). In this area, high levels of As and Cd were recorded in the kidneys of mice, which suggested the great bioavailability of these compounds. According to these authors, in already-known polluted areas, biomonitoring is a more powerful strategy, since it can provide valuable information about the bioavailability of these hazards, and the consequent impact on biota (Talmage and Walton 1991).

Overall, there is a pattern of similarity between the geological and soil chemical analysis studies over the country and the present biomonitoring study. Thus, it becomes evident that the hedgehog (as a bioindicator) and the levels of metal(loid)s in its organs clearly reveal the bioavailability of these elements in these areas. Under a One Health approach, it is realistic to believe that similar regional differences could be observed in other animal and human populations. Therefore, future monitoring plans for each metal(loid), as well as mitigation strategies in the most critical areas, such as phytoremediation (Gascó et al. 2019; Martínez-López et al. 2014), should be considered to avoid the health effects associated to these hazards. Notwithstanding, some regions from the present study were only represented by one or two hedgehogs, which is a limitation to a precise interpretation and statistical comparison of the results between regions. Moreover, it is not possible to guarantee the presence of all the age groups in every location, which maybe also conditioning our results.

### Regression between spines and internal tissues

The spines and hair are mainly composed by keratin, a protein that contains sulfhydryl, which can bind several metal(loid)s. The contact of each hair unit (or spine) with the bloodstream at the follicle allows the incorporation metal(loid)s in circulation during the spine’s growth (Beernaert et al. 2007; Jota Baptista et al. 2022). However, a standard methodology for the use of these samples in wildlife (*e.g.* during the sample preparation, as washing or drying procedures) has not been established yet and sometimes is not referred by the authors, which may lead to discrepant results and different conclusions regarding the correlations between skin coating and internal concentrations. In the present study, ultrasounds washing and oven dry were used, and only Co and Ni showed statistically significant values of the regression coefficients between spines and one or both internal tissues. On the other hand, wood mice (*Apodemus sylvaticus*) hair showed positive and significant correlations for Cd (with liver, lungs, muscle and kidneys) and for Pb (with liver and kidneys) (Beernaert et al. 2007). In bats, strong relationships were reported between the Cd, Cu and Pb detections in hair and in the kidneys, liver, stomach content and bones (Hernout et al. 2016). In Iberian wolves, significant correlations were found between hair and liver Pb concentrations and between hair and kidney Cd concentrations (Hernández-Moreno et al. 2013). In this last case, the hair cleaning was performed with acetone (three washing cycles of 10 ml), a different method from the one used in the present study.

Considering only hedgehogs’ studies, D'Havé et al. (2005); D’Havé et al. (2006) reported significant correlations with the liver and kidneys for all metal(loid)s except for Ag, Al, Fe, Ni and Zn. Moreover, significant relationships between spines and muscle were found for Cd, Co, Cr, Cu and Pb. Nevertheless, like in the present study, for some metal(loid)s, the regression only explains a small variation in tissue concentration, as indicated by the small *R* or *R*^2^ values. These authors presented some differences between hair and spines, even though spines are modified hairs (with the same keratin composition). Vermeulen et al. (2009) reported significant relationships between spines and blood for As, Cd, Cr and Pb. However, these authors also mentioned that many factors influence the extent to which metal(loid)s bound to hair and spines, leading to differences in measured concentrations (Vermeulen et al. 2009). Spine moulting is known as a continuous process, though some studies have been suggesting that hedgehogs may undergo periodic partial moults (D'Havé et al. 2005; D’Havé et al. 2006; Reeve 1994), affecting the incorporation of metal(loid)s, and which may explain the differences found between studies.

## Conclusions

Globally, except for Cu and Cd, samples of hedgehogs did not present alarming metal(loid) results. Nevertheless, mostly due to anthropogenic activities, some regions showed elevated concentrations of some metal(loid)s that should be considered for future monitoring plans and mitigation strategies. Sex does not seem to significantly affect the metal(loid)s’ concentrations. Heavier hedgehogs tend to present higher levels of meta(loid)s, presumably due to a continuous food intake and accumulation of these substances in the adipose tissue. Overall, adult hedgehogs presented higher levels of metal(loid)s, which expresses a long-term exposure and accumulation of these compounds in their tissues. According to the regression results, spines are not suitable indicators of every metal(loid)s. Further research (*e.g.* using other species; including animals from every district; or using more tissues) is necessary to establish which areas should be prioritized for each metal(loid) and eventually apply the necessary mitigation strategies (for instance, phytoremediation) to avoid the One Health consequences (such as soil and plant composition changes or multiple disorders in animals and humans).
